# Editorial: Transforming neurological recovery: the promise of regenerative neurorehabilitation

**DOI:** 10.3389/fneur.2026.1812150

**Published:** 2026-04-30

**Authors:** Stefania Dalise, Claudia Alia, Carmelo Chisari, Federico Cremisi, Michel Modo

**Affiliations:** 1 Unit of Neurorehabilitation, Department of Neuroscience, University Hospital of Pisa, Pisa, Italy; 2 Neuroscience Institute, National Research Council (CNR), Pisa, Italy; 3 Department of Translational Research and New Technologies in Medicine and Surgery, University of Pisa, Pisa, Italy; 4 Laboratorio di Biologia, Scuola Normale Superiore, Pisa, Italy; 5 Department of Radiology, University of Pittsburgh, Pittsburgh, PA, United States; 6 Department of Bioengineering, University of Pittsburgh, Pittsburgh, PA, United States

**Keywords:** biomarkers, molecular regulation, neural stem cells, neuroplasticity, neurorehabilitation, regenerative medicine, spinal cord injury

**Figure 1:**
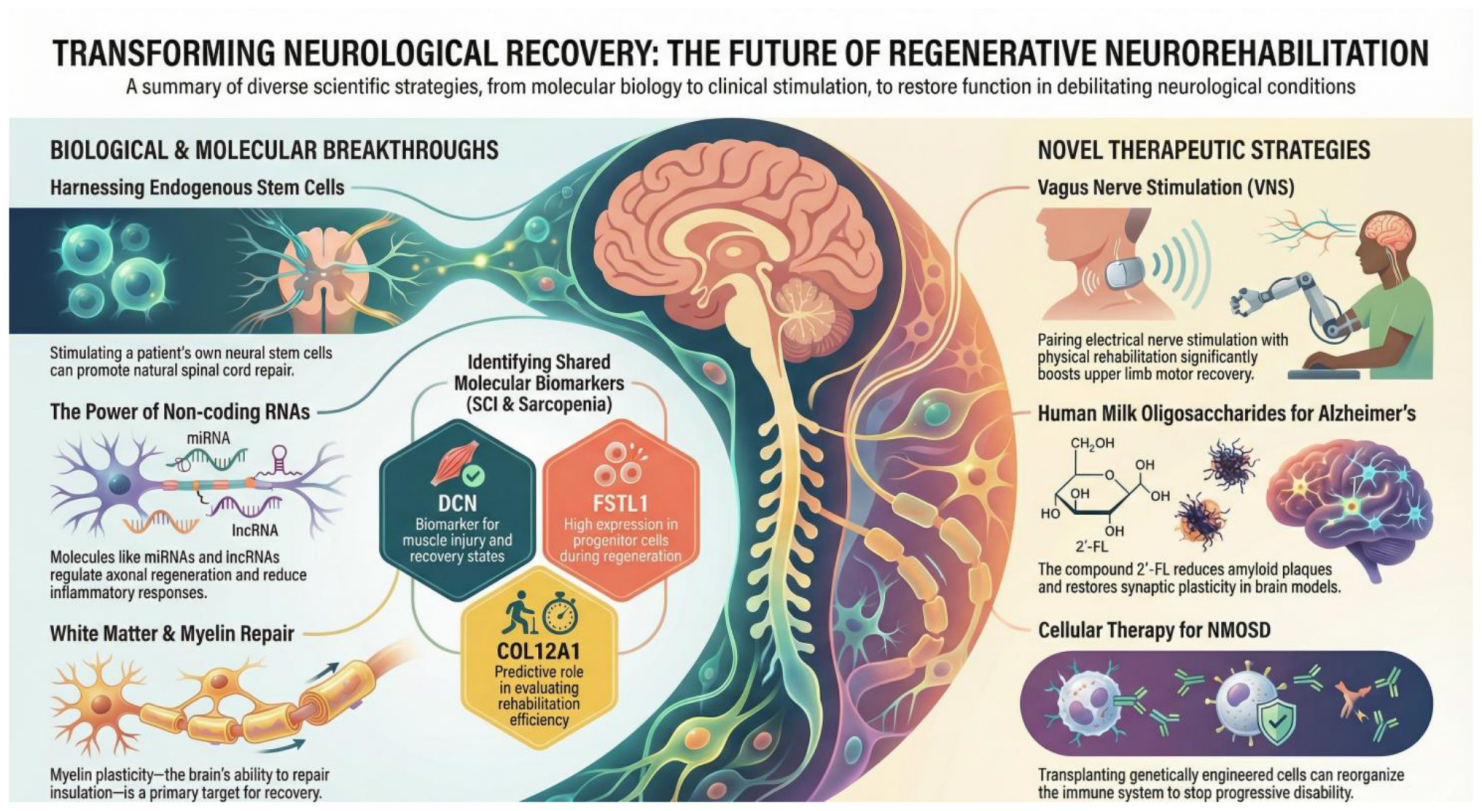
Conceptual framework of regenerative neurorehabilitation. Schematic overview illustrating the integrated mechanisms underlying regenerative neurorehabilitation across neurological disorders. The figure highlights the interplay between cellular therapies, endogenous neural stem cells, oligodendrocyte precursor cell-mediated myelin repair, molecular regulation (including non-coding RNAs, inflammation, and oxidative stress), neuromodulation through vagus nerve stimulation, and systemic factors, such as muscle regeneration and sarcopenia. Together, these interconnected pathways emphasize how multimodal, activity-dependent, and biologically informed rehabilitation strategies converge to promote neural repair, plasticity, and functional recovery.

Functional recovery following injuries or diseases of the central nervous system (CNS) remains one of the most formidable challenges in modern medicine, largely due to the tissue's limited intrinsic repair capacity. This Research Topic, entitled “*Transforming neurological recovery: the promise of regenerative neurorehabilitation*,” explores the convergence of regenerative medicine and neurorehabilitative strategies, illustrating how molecular, cellular, and physical approaches can be integrated to promote meaningful neurological recovery (Figure 1).

A substantial part of this Research Topic focuses on spinal cord injury (SCI), a condition in which endogenous repair is constrained by a complex inhibitory microenvironment. Several contributions highlight the potential of endogenous neural stem cells (eNSCs) as an intrinsic reservoir for regeneration (Wang T. et al.). Although these cells proliferate after injury, their differentiation is biased toward astrocytic lineages, limiting neuronal replacement. Understanding the developmental signaling mechanisms and microenvironmental cues governing eNSC fate is therefore critical for designing multimodal strategies—combining pharmacological, biomaterial, and rehabilitative approaches—to enhance neuronal regeneration.

Complementing these cellular perspectives, regulatory roles of non-coding RNAs (ncRNAs) are presented as an additional molecular layer influencing secondary injury processes (Bao et al.). By modulating inflammation, oxidative stress, and axonal remodeling, ncRNAs emerge as promising therapeutic targets for shaping the post-injury environment and supporting repair. Extending this molecular framework, comprehensive genomic analyses reveal shared biomarkers between SCI and sarcopenia, identifying hub genes with potential diagnostic and prognostic value (Wang B. et al.). These findings underscore the systemic dimension of SCI and suggest that genomic signatures may help monitor rehabilitation efficacy and guide personalized interventions.

White matter plasticity represents another central theme of regenerative neurorehabilitation. Unlike neurons, white matter exhibits a measurable capacity for repair through oligodendrocyte precursor cells (OPCs), which can be recruited to remyelinate axons in response to functional demands (Baldassarro et al.). By integrating clinical imaging data with preclinical evidence, this Research Topic highlights how targeted rehabilitation and physical activity may activate quiescent OPCs and enhance endogenous myelin repair, reinforcing the concept that activity-dependent mechanisms are key drivers of structural recovery.

Translational innovation is further exemplified by a pilot clinical trial, which employed a randomized controlled protocol, to evaluate vagus nerve stimulation (VNS) paired with intensive upper limb rehabilitation in individuals with chronic cervical SCI (Yozbatiran et al.). This neuromodulatory approach seeks to amplify neuroplasticity during task-specific training, offering a promising avenue to improve motor outcomes, whilst demonstrating how bioelectronic interventions can be integrated into regenerative rehabilitation paradigms.

Beyond traumatic injury, the scope of this Research Topic extends to neuroimmune and neurodegenerative disorders. In neuromyelitis optica spectrum disorder (NMOSD), cellular therapies are explored as potential disease-modifying strategies capable of reorganizing immune responses to provide long-term stabilization that goes beyond conventional immunosuppression (Zhong et al.). In parallel, preclinical studies in Alzheimer's disease models highlight the neuroprotective properties of 2′-fucosyllactose, a human milk oligosaccharide. This intervention reduced amyloid pathology, attenuated neuroinflammation, and restored synaptic plasticity to improve cognitive performance (Munni et al.). These findings point to the potential role of metabolic and nutritional interventions within broader regenerative frameworks for neurodegenerative conditions.

In conclusion, the entirety of articles in this Research Topic emphasizes the multifactorial nature of neurological recovery and advocates for integrated, personalized therapeutic strategies. By bridging molecular biology, cellular regeneration, neuromodulation, and activity-based rehabilitation, this Research Topic moves the field closer to a paradigm in which recovery is driven not only by compensation, but by genuine biological repair. We anticipate that these contributions will stimulate interdisciplinary collaboration and accelerate the translation of regenerative neurorehabilitation advances into clinical practice, emphasizing that the synergy between biological repair mechanisms and activity-dependent rehabilitation represents a critical pathway toward meaningful neurological recovery.

